# Supplementary material for: Removing facial features from structural MRI images biases visual quality assessment
Source: PLoS Biol. 2025 Apr 30;23(4):e3003149. doi: 10.1371/journal.pbio.3003149 (PMC12143891; doi:10.1371/journal.pbio.3003149)
Supplement: Attachment — Submitted filename: PBIOLOGY-D-24-02964R2-response_letter.pdf [file pbio.3003149.s014.pdf]

Authors' Response to Reviews of

C. Provins et al. – "Defacing biases in manual and automated quality assessments of structural MRI with MRIQC"

AR: Authors' Response

We thank the Reviewer and the Editors for the careful revision, the valuable feedback, and the time dedicated to our manuscript.

In this response, we reproduce all changes made to our submission, marked up with the *Edit* keyword. Page and line numbers of the "Original" text cross-reference the submitted version **without** markup of tracked changes. Page and line numbers of the "Revised" text cross-reference the new document **with** markup of tracked changes. For example:

Edit: **Original (p. N, ll. aaa–bbb):**

> Text in the ~~submitted~~ manuscript—version with~~out~~ track-changes markup,—with ~~deletions marked up by a strike-through line~~.

Revised (p. M, ll. yyy–zzz):

> Text in the revised manuscript—version with track-changes markup,—with additions marked up in blue-color text.

This response letter is structured as follows. First, we introduce a General comment. Then, we include a response to the reviewers' comments point-by-point in the Reviewer #1 section. Finally, we have also indicated Minor corrections not prompted by the reviewer.

General comment

Prompted by comment R#1.11 requesting more clarity in the definition of "bias" at l. 421, we carried out a thorough revision of our Bland-Altman (BA) analysis and related nomenclature. In doing so, we discovered a misinterpretation within our Stage 1 IPA registration that had, unfortunately, escaped all scrutiny. Bland & Altman—refs. [37, 38] in the manuscript—proposed evaluating the disagreement of measurement methods using the 95% range of limits of agreement (LoA) of their differences. In addition, they proposed testing the significance of estimated nonzero biases by evaluating the 95% confidence interval (CI) of the mean of those differences.

To address these inconsistencies, we thoroughly revised the text to ensure that the definitions of bias, LoA, and CI are aligned with Bland & Altman's recommendations at the earliest mention of BA analysis in the Methods section. Our revisions ensure these three quantities (*bias*, *LoA*, and *95% CI for the bias*) are no longer conflated or misstated within the text. Additionally, we had originally stated (ll. 234–235) that we would apply a non-parametric approach to compute the 95% CI, but in other sections of the manuscript, we referred to a parametric version. In the revised manuscript, we report both parametric and non-parametric (bootstrap) 95% CIs in order to safeguard against

potential non-normal distributions of ratings. We correct these elements at the appropriate points (see p. 7, ll. 225–235 and Section S6.2 in the Supplementary Materials).

Edit: **Original (p. 7, ll. 225–235):**

> **Confirming that on average ratings are higher on defaced images.** We used Bland-Altman (BA) plots [37] to visualize the bias and the ~~limits of~~ agreement of manual quality ratings between the nondefaced and the defaced condition. Five BA plots were generated (Fig. 2), one for each individual rater and one pooling the ratings from all raters together. We used the individual-rater BA plots to investigate whether the bias varied with respect to the quality grade attributed and how the bias changes depending on the rater. ~~The BA plot with the pooled ratings was used to test the significance of the bias.~~ To demonstrate that ~~the ratings of the defaced condition were higher than the corresponding ratings on the nondefaced condition,~~ we tested whether the bias was significantly negative. ~~Biases in the BA plot were deemed significant if the zero-difference fell outside the 95% limits of agreement. Because the distribution of ratings was not Gaussian (as per a Shapiro-Wilk test), we used non-parametric 95% limits of agreement [38].~~

Revised (pp. 8–9, ll. 241–271):

> **Confirming that—on average—ratings are higher on defaced images.** We used Bland-Altman (BA) plots [37] to visualize the bias and the agreement of manual quality ratings between the nondefaced and the defaced conditions. Five BA plots were generated (Fig. 2), one for each individual rater and one pooling the ratings from all raters together. We used the individual-rater BA plots to investigate whether the bias varied with respect to the quality grade attributed and how the bias changes depending on the rater. [m]The bias $\mathbb{E}[\Delta_{\text{ndef-def}}]$ is defined [37] as the mean of rating differences $\Delta_{\text{ndef-def}} = r^s_{\text{ndef}} - r^s_{\text{def}}$, where $r^s_{\text{condition}}$ are the ratings corresponding to the nondefaced (ndef) and defaced (def) conditions of a single subject's image s. Therefore, to investigate our hypothesis that humans' *ratings are higher on defaced images*, we confirmed that $\mathbb{E}[\Delta_{\text{ndef-def}}]<0$ for all raters, aggregated and individually (Fig. 2). Bland & Altman [37] recommend assessing the agreement between measurements by calculating the 95% limits of agreement (LoA), defined as *the range in which 95% of the differences $\Delta_{\text{ndef-def}}$ are expected to fall*. The wider the 95% LoA, the worse the agreement between conditions, implying lower reliability. In practice, *two measurement approaches cannot be used interchangeably if their LoA are too broad*. Following [37], LoA are calculated as follows:
>
> $$\text{LoA} = \left(\mathbb{E}[\Delta_{\text{ndef-def}}] - z_{\alpha/2}\,\sigma, \mathbb{E}[\Delta_{\text{ndef-def}}] + z_{\alpha/2}\,\sigma\right),$$
>
> where σ is the sample standard deviation, and $z_{\alpha/2}$ is the critical value—with $z_{\alpha/2}$=1.96 for the 95% coverage range of the differences, and $z_{\alpha/2}$=2.0 recommended as a close approximation [37]. To demonstrate that nonzero biases were not obtained by chance, we tested whether the bias was significantly negative by checking if $\Delta_{\text{ndef-def}}$=0 was contained within the 95% confidence interval (CI) for the bias [37]. We calculated the 95% CI for the bias under the assumption of normality and reasonably large sample sizes following Bland & Altman's indications [37], with:
>
> $$\text{CI} = \left(\mathbb{E}[\Delta_{\text{ndef-def}}] - 1.96\frac{\sigma}{\sqrt{n}}, \mathbb{E}[\Delta_{\text{ndef-def}}] + 1.96\frac{\sigma}{\sqrt{n}}\right),$$
>
> where n is the sample size. To protect from violations of the normality assumption, we also computed them via bootstrapping [38]. We reported both estimates and tested for significance

with the more conservative option. Additional considerations on the interpretation of BA plots, the 95% LoA, and the 95% CI of the mean bias are provided in the Supplementary Materials, subsection S6.2.

We have updated Supplementary Table S2, including the 95% CI of the bias for the analysis of manual ratings. All statistics derived from BA plots for both analyses (human ratings and IQMs) are made available through the GitHub repository indicated in the Data and Software Availability statement[1]. These tables also contain the nonparametric calculation of 95% CIs demonstrating that they are all very close to the parametric counterpart without exception. Finally, we also updated Figure 2 to report the sample size, the 95% LoA, the bias, and the corresponding 95% CI for each BA plot (presented below in response to Comment R#1.11).

These changes have also been reflected as an additional protocol deviation marked with superscript "m", causing the update of former superscript "m" into "n":

Edit: **Original (p. 10, l. 317):**

> [m]Two IQMs, Mortamet [17] "quality index 1" and the "5th percentile of the background intensity",

Revised (p. 11, ll. 354–359):

> [m]**Corrected interpretation of BA plots**. Our pre-registered plan employed 95% LoA of the difference and 95% CI of the bias as interchangeable terms, which is incorrect. Our Stage 2 report was updated to employ unambiguous nomenclature for the bias $\mathbb{E}[\Delta_{\text{ndef-def}}]$, included the definition of LoA and CI as proposed initially by Bland & Altman [37, 38] and provided further details regarding the interpretation of LoA and the significance of the biases.
>
> [n]Two IQMs, Mortamet [17] "quality index 1" and the "5th percentile of the background intensity",

Edit: **Original (p. 7, l. 237):**

> was evaluated based on 58~~[m]~~ out of 62 IQMs calculated

Revised (p. 9, l. 273):

> was evaluated based on 58[n] out of 62 IQMs calculated

Despite this confusion, the interpretation of the results remains unchanged because a significant bias means that the 95% CI for the mean difference excludes zero, implying that the true mean difference is unlikely to be zero. Bland & Altman stressed that judging relevance depends on how large that mean difference is compared to clinical or practical standards, not merely whether it is statistically different from zero. Therefore, our original interpretation, comparing the bias with the LoA and the average magnitude of the measure, is a good proxy for the relevance of the bias. To clarify this, we have added the following text on the interpretation of BA plots in section S1.1 of the Supplementary Materials:

Edit: **Original (no text to show):**

[1]Under the path TheAxonLab/defacing-and-qc-analysis/outputs/

Revised (Supplementary Materials, p. 3, Section S1.1):

> **Interpretation of BA plots, LoA, and CI of the bias.** Bland & Altman indicated that even though a bias may not be significant, that doesn't mean that the bias is clinically irrelevant. For example, if the bias between two blood pressure devices is 1.5 mm Hg with a 95% CI of –0.2 to 3.2, since zero is within this interval, there is no significant bias. The 95% LoA might be –5.0 to 8.0 mm Hg, meaning most measurements differ by at most about 5 to 8 units. A clinician will judge if that range is acceptable. The reverse situation may also be true: a significant bias can be clinically irrelevant, which is what occurred to the vast majority of IQMs (Figure S16). Suppose the two devices showcase a bias of 3 mm Hg, with 95% CI of 2.999 to 3.001 mm Hg—i.e., significant bias,—and a 95% LoA of 2.95–3.05. The two devices are interchangeable despite the significant bias because they agree substantially—provided the 0.1 mm span of LoA is not clinically meaningful. Indeed, to use one or another, we would need to account for the 3 ± 0.001 mm Hg systematic bias between the two devices.

In our case, and more remarkably for the IQMs' analysis because of the much larger sample size, all IQMs except "efc" showed a bias significantly different from zero (albeit very small), as a result of extremely narrow CIs deriving from the large sample size, but also narrow 95% LoA, indicating relatively small variability introduced by defacing. The reviewer specifically asked a related question in comment R#1.2, where this particular point is examined in detail.

In editing the text, we noticed that we had inadvertently changed reference [38] with respect to the Stage 1 document. Unfortunately, this error was concealed by the change from APA citations—which show the year—to the journal's style where the year is not present. We have restored [38] back to the reference found in the pre-registered Stage 1 document. This change was relevant because Bland & Altman introduced testing the significance of the bias through bootstrapping in their 1999 paper.

Edit: **Original (p. 21, ll. 698–699):**

> 38. Bland JM, Altman DG. ~~Statistical methods for assessing agreement between two methods of clinical measurement. The Lancet. 1986;327(8476):307–10.~~

Revised (p. 25, ll. 799–801):

> 38. Bland JM, Altman DG. Measuring agreement in method comparison studies. Statistical Methods in Medical Research. 1999;8(2):135-160.

Finally, we deemed it necessary to make a more explicit mention to the LoA estimated for the raters:

Edit: **Original (p. 13, ll. 413–414):**

> ~~T~~hese plots also reflected that raters were not equally affected by defacing.

Revised (p. 14, ll. 455–457):

> Although all raters displayed individual LoA larger than one unit indicating that defacing introduced large variability, these plots also reflected that raters were not equally affected by defacing.

1. Reviewer #1

R#1.1:

This work investigates if there are differences in quality ratings of T1w MRI before and after de-facing, a step commonly applied to de-identify images before wider sharing. The analysis has been performed following a PCI Registered Report. The work seems to have been carried out in accordance with the pre-registered methodology and any deviations clearly documented and results mainly well presented. I think that the manuscript should be published but suggest a few additions and changes that I think might improve the readability and help readers understand the findings more generally.

AR: We thank the reviewer for their valuable feedback and we have revised the manuscript in light of all the suggested additions and changes.

R#1.2:

My take home from the findings is that there are no differences in automated QC measures (using MRIQC) between de-faced and non-defaced images. Except for (@L535) entropy-focus criterion. I think to help readers understand this finding, reiterate the difference in efc is only 1 IQM out of <X total no.> of QC measures derived and tested. And provide more on what some of the other IQMs are that are not different between the defaced and non-defaced conditions - i.e. I would imagine snr_gm and cnr are likely to be more important QC measures to most readers - so some context here would help.

AR: We thank the reviewer for this effort in summarizing our findings about MRIQC's metrics. Based on this summary, we can confirm that the paper is clear in this regard. Nonetheless, we appreciate the suggestion that the contrast of a single IQM versus a total of 58 we finally analyzed (64 generated by MRIQC, of which six were dropped as indicated on p. 10, ll. 317–320). We also agree that we need to "provide more on what some of the other IQMs are that are not different between the defaced and nondefaced conditions", explicitly mentioning `snr_gm` and `cnr`, as well as also talking about `snrd_*`. We have edited the Discussion section accordingly at the point indicated by the reviewer (l. 535) and added one new citation [48] to support the argument.

Edit: **Original (p. 17, ll. 535-536):**

> In a post-hoc exploration using BA plots, ~~we identified the entropy-focus criterion ("efc") as the single metric that differed significantly after defacing.~~ Our ~~conclusion~~ contrasts with [5] (...)

Revised (pp. 19–20, ll. 613–628):

> In a post-hoc exploration using BA plots, only the "efc" metric showed a meaningful difference between conditions out of the total 58 IQMs investigated. Although most IQMs had significant biases (Fig. S16) due to the large sample size, these biases were minimal, and their LoA were narrow in comparison to their corresponding variation ranges. Conversely, "efc" was the only metric displaying a broad LoA compared to its typical range (Fig. S16). In addition, except for "efc", no other IQM's LoA excluded the zero-difference line, indicating that very rarely differences in "efc" will be small between conditions. This result seems a consequence of

MRIQC's definition of most metrics, such as contrast-to-noise ratio ("cnr") or signal-to-noise ratio in the gray matter ("snr_gm"), which are extracted from areas within the brain and, therefore, unaffected by defacing. The "efc" metric is an exception among IQMs including background information in their definition. For example, the "snrd_*" family of metrics is akin to their "snr_*" counterparts with the difference that the signal variability is estimated in the background [48]. While "efc" employs all the background surrounding the head and neck, "snrd_*" metrics discard background information outside of a "hat" mask that excludes the background below the nasio-occipital plane. Hence, they are also insensitive to the area altered by defacing. Our results contrast with [5] (...)

Adding the new citation renumbered the last reference by one:

Edit: **Original (p. 22, l. 719):**

48. Pizarro RA, Cheng X, ...

Revised (p. 26, ll. 821–824):

48. Dietrich O, Raya JG, Reeder SB, Reiser MF, Schoenberg SO. Measurement of SNRs in MR images: influence of multichannel coils, parallel imaging and reconstruction filters. JMRI. 2007; 26(2):375—385.

49. Pizarro RA, Cheng X, ...

To note, the discussion about the "efc" (as well as the definition of the acronym) was introduced previously in the Results, which we have updated consistently:

Edit: **Original (p. 14, ll. 460–463):**

In a post-hoc exploration using BA plots (Fig. S16), we found that ~~only the entropy-focus criterion ("efc") presented a significant bias between the nondefaced and defaced images. This bias is also clearly visible in the~~ violin plots (Fig. S14).

Revised (pp. 16–17, ll. 521–529):

In a post-hoc exploration using BA plots (Fig. S16), we found that nearly all IQMs showcased very narrow 95% LoA and minimal biases—yet nonzero—compared to their variation ranges. The narrow LoA indicate that the variance introduced by defacing into the IQMs is relatively small. Biases were deemed significant in most of the IQMs, which immediately follows the relatively large sample size. Only the entropy-focus criterion ("efc") emerged as an exception, showcasing a substantial bias and a broad 95% LoA, which, in addition, did not contain zero. As opposed to all other IQMs, the bias corresponding to the "efc" was also clearly noticeable in violin plots (Fig. S14).

R#1.3:

And if essentially there is no difference in automated QC measures by defacing, I don't agree with the recommendation in the Discussion starting on L481 "Since defacing..." which says it's important to report results from the non-defaced data? - but why, when it's essentially no

> *different to the de-faced data? If this specifically relates to the manual rating please see comment on this below.*

AR: We thank the reviewer for making us aware that our manuscript is lacking critical clarity in describing this interpretation of the results. As the reviewer anticipates, our recommendation stems from the bias found in manual ratings. We have clarified in the Discussion that our recommendation to report non-defaced data was based on manual rater bias rather than the automated QC results. Specifically, our recommendation when data are to be openly shared—and anatomical images defaced—is that the visual reports of *MRIQC* be obtained before defacing to ensure human raters are not biased, and those reports be shared with the published repository so reusers can perform QA/QC in the closest conditions to those of the original authors.

Edit: **Original (p. 15, ll. 480–486):**

> This indicates that human raters are sensitive to defacing, possibly more for more experienced raters. ~~Since defacing is necessary to protect participants' privacy, our work shows the importance of including~~ QA/QC materials ~~(such as MRIQC's visual reports)~~ extracted from the original (nondefaced) images ~~when sharing defaced data~~, as long as the participant's identity cannot be reconstructed from those additional resources. This will enable data re-users to perform QA/QC ~~in more adequate settings~~, minimizing the risk of artifacts going unnoticed and biasing analyses.

Revised (p. 18, ll. 552–564):

> This indicates that human raters are sensitive to defacing, possibly more for more experienced raters. The first implication of this result is that QA/QC protocols establishing a visual checkpoint on the unprocessed data—e.g., with *MRIQC*'s visual reports [23]—should generate visualization and other QA/QC materials before defacing anatomical images. Defacing is generally required for compliance with applicable privacy protection regulations if data are to be processed on systems with low-security clearances or data will be made accessible to third parties or publicly. Regardless of the motivation to deface anatomical images, our results support the recommendation of providing manual QA/QC materials extracted from the original (nondefaced) images alongside the defaced dataset, as long as the participant's identity cannot be reconstructed from those additional resources. This will enable data re-users to perform QA/QC in settings more consistent with those of the original authors', minimizing the risk of artifacts going unnoticed and biasing analyses.

R#1.4:

> *In the concluding sentences it's stated that you've shown how defacing can "degrade data reusability" but I'm not sure you have? And again I don't think the conclusion that "We argue that sharing QA/QC materials from nondefaced images can help mitigate these issues while ensuring participant privacy" is the correct conclusion based on the results which show that for automated QC defacing doesn't impact results.*

AR: We thank the reviewer's candor for pointing out our oversight. Indeed, we have not articulated this conclusion properly. To comprehensively address this comment, we have better contextualized our work at the beginning of the discussion and have reformulated the conclusion. The discussion has

been edited as follows:

Edit: **Original (p. 15, ll. 474–475):**

> We presented a registered report investigating the alterations introduced by defacing in the quality assessment of manual and automatic ratings of T1w whole-brain images.

Revised (p. 17, ll. 540–546):

> Typical neuroimaging workflows involve many image-processing steps, contributing to analytical variability that cascades throughout the pipeline. Even initial processing steps considered relatively innocuous, such as reorienting the volume to align the anterior-posterior commissure line with the axial plane, may conceal relevant design decisions and inadvertently introduce variability. Because defacing has been shown to influence downstream processing [1,4], this registered report investigated the alterations introduced by this step in the manual and automatic QC of whole-brain T1w images.

The conclusion has been modified as follows:

Edit: **Original (p. 17, 554–560):**

> Our report showed that defacing influences human quality assessments of T1w images, with lower-quality nondefaced images likely rated higher after defacing. *MRIQC*'s automatically-extracted image quality metrics (IQMs), on the other hand, remained largely unaffected, as the tool does not consider areas typically altered by defacing when computing the IQMs. We show how privacy protection measures, like defacing, can degrade data reusability and emphasize that actions early in the computational pipeline may have significant downstream effects. We argue that sharing QA/QC materials from nondefaced images can help mitigate these issues while ensuring participant privacy.

Revised (p. 20, ll. 649–658):

> The initial processing steps in the computational pipeline may have relevant downstream effects. Our report showed that defacing influences human quality assessments of T1w images, with lower-quality nondefaced images likely rated higher after defacing. Inadequate manual QC decisions deriving from visualization of defaced images could undermine the soundness of results. *MRIQC*'s automatically-extracted IQMs, on the other hand, remained largely unaffected, as the tool does not consider areas typically altered by defacing when computing the IQMs.

R#1.5:

For the manual ratings - my understanding is that the raters saw the whole image, so it's immediately obvious if the image is a defaced one or not? Wouldn't it be the case then that the raters, end up focusing on IQ issues related to those around the face - e.g. ghosting from the eyes - but really what's important is the IQ of the brain? Making the assumption that most research will be using the brain data. And then probably skull stripping in their analysis. Then maybe focusing on the IQ of the brain becomes overlooked and raters, consciously or not, focus on the face area? What I'm wondering is that maybe non-defaced images are simply marked

> *to have worse quality due to eye motion, but that's not really a good measure of overall IQ for researchers who want to study the brain. This would be easy to check - if the raters mainly specified "eye spillover. . . " in the rating widget when the de-faced images were scored better? I think this should be reported and discussed. And if this is true, I think findings about differences in manual QC should be down-played a little.*

AR: We thank the reviewer for this comment.

Regarding the first question—indeed—the raters could immediately figure out whether they were rating a defaced or a nondefaced case because they employed *MRIQC* visual reports, as described in the introduction (p. 4, ll. 134–135).

The second question from the reviewer: "Wouldn't it be the case then that the raters, end up focusing on IQ issues related to those around the face - e.g. ghosting from the eyes - but really what's important is the IQ of the brain?" reveals a widespread misconception about MRI quality that our manuscript fails to clarify as it stands. The short answer is "no": even if a study focuses on the brain only, those researchers must be careful about artifacts elsewhere. The reason is that MRI images are the result of *reconstructing* the spatial information from data recorded in the frequency domain. As a result, imaging artifacts are rarely confined within a region of the image. For example, in an image with a wrap-around artifact along the anterior-posterior axis, the skull of the back of the head could overlap the prefrontal cortex. When the image is complete, even an inexperienced rater will find it easy to follow the skull contour and spot its overlap with the cortex. However, if the brain has been extracted and the head is removed, this overlap will be elusive to the observer regardless of their experience. **We have added Figure S22 to the Supplementary Materials with this particular example.** The "eye spillover" example is also consistent with this explanation, and will be addressed later below.

Because the manuscript should be unequivocal about how the raters were instructed to screen the data, we have updated it accordingly:

Edit: **Original (p. 5, ll. 139–141):**

> To ensure consistency of their training, raters read our published QC protocol [23] and took a 3h[g] training session. At the end[g] of this session, raters self-assessed their experience as either beginner, intermediate, or advanced.

Revised (p. 5, ll. 154–159):

> To ensure consistency of their training, raters read our published QC protocol [23] and took a 3h[g] training session. Following our guidelines [23], raters were instructed that it was critical to scrutinize the background visualization carefully because most of the artifacts are more noticeable in the absence of a signal of interest. Several examples supported this remark during the training session (see Fig.1, Supplementary Fig.S22, and the training materials). At the end[g] of this session, raters self-assessed their experience as either beginner, intermediate, or advanced.

Finally, we want to address the comment about "eye spillover" initiated here and later referenced in comment R#1.7. First, we have clarified the definition of the artifact with its introduction in the caption of Figure 1 (see R#1.7 for the specific edits). Second, we have eliminated it as an example

in the related discussion in ll. 542–545, and at the same time, addressed this misconception by improving the justification provided by reference [49] ([48] in the submitted version), and a new reference [50] to our previous work on functional MRI about this particular topic:

Edit: **Original (p. 17, 542–545):**

> Based on our findings, we recommend that *MRIQC* (and any QA/QC tool for unprocessed T1w images) include metrics sensitive to artifacts in areas typically removed or altered by defacing ~~[48], such as eye spillover.~~ These metrics should be clearly ~~identified~~ and excluded from analyses involving defaced images where they are not ~~available~~.

Revised (p. 20, ll. 634–640):

> Based on our findings, we recommend that *MRIQC* (and any QA/QC tool for unprocessed T1w images) includes metrics sensitive to artifacts in areas typically removed or altered by defacing. Prior work by Pizarro et al. [49] on anatomical MRI and our later work on functional MRI [50], justified how information "outside" the brain region is relevant to assess the quality of imaging "within" the region of interest. These metrics—e.g., those proposed in [49] and derivations thereof—should be clearly annotated and excluded from analyses involving defaced images where they are not applicable.

Edit: **Original (p. 22, ll. 719–724):**

> 48. Pizarro RA, Cheng X, Barnett A, Lemaitre H, Verchinski BA, Goldman AL, et al. Automated Quality Assessment of Structural Magnetic Resonance Brain Images Based on a Supervised Machine Learning Algorithm. Front Neuroinf~~ormatics [Internet]~~. 2016 ~~[cited 2017 Jan 12]~~;10. ~~Available from: http://journal.frontiersin.org/article/10.3389/fninf.2016.00052/abstract~~

Revised (p. 26, ll. 824–831):

> 48. [See reference added in Comment R#1.2]
>
> 49. Pizarro RA, Cheng X, Barnett A, Lemaitre H, Verchinski BA, Goldman AL, et al. Automated Quality Assessment of Structural Magnetic Resonance Brain Images Based on a Supervised Machine Learning Algorithm. Front Neuroinf. 2016; 10.
>
> 50. Provins C, Markiewicz CJ, Ciric R, Goncalves M, Caballero-Gaudes C, Poldrack RA, Hagmann P, Esteban O. Quality control and nuisance regression of fMRI, looking out where signal should not be found. In: Proc. Intl. Soc. Mag. Reson. Med. 31 (ISMRM), 2022; pp. 2683, London, England, UK.

For convenience, this is the new figure added to the Supplementary materials [full resolution version there]:

Edit: **Original (Supplementary Materials):**

> [no figure]

Revised (Supplementary Materials, pp. 30–31):

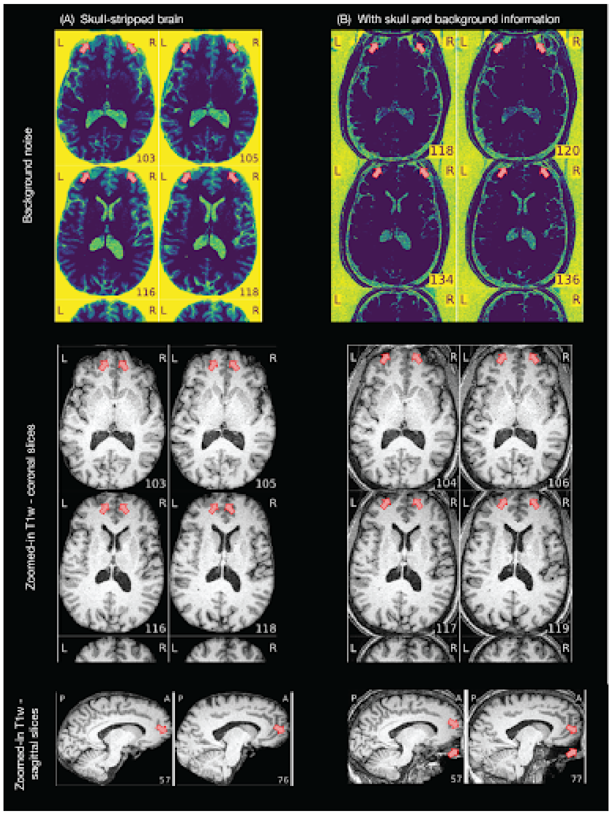

S21 Figure. Screening the background helps detect artifacts within the brain. This figure (next full page) illustrates how including information outside the brain outline improves artifact detection, particularly for wrap-around artifacts. In this example, the posterior of the head is “wrapped around” and aliases over the prefrontal cortex, compromising the signal in that region. When the brain has been extracted (A), the overlap of the skull becomes elusive. Conversely, when the assessment is performed before brain extraction (B), the overlap is easily traceable, regardless of the observer’s experience. All images are screenshots from MRIQC visual reports extracted from the T1w scan of subject “10377” from the Consortium for Neuropsychiatric Phenomics dataset (Gorgolewski, Durnez, and Poldrack 2017).

R#1.6:

"QA/QC" used throughout - then sometimes QA and sometimes QC on its own. Are they intended to be used interchangeably or as separate things - if interchangeably only one term is needed, if separate, explain.

AR: We thank the reviewer for mentioning this interesting discussion. Indeed, the terms are not interchangeable: First, Quality Assurance (QA) refers to protocols, decisions, and operations to keep the quality levels—of images—above pre-specified (quantitative or qualitative) levels. Second, Quality Control (QC) is a more specific term to refer to those actions taken when images do not meet the necessary criteria for inclusion in a study. Unfortunately, the line between QA and QC is often

defined by the eyes of the beholder. Most QA actions are triggered by QC decisions: for example, if a researcher finds an electromagnetic spike in one image, that case will be discarded from the study (QC decision). However, it is critical that the MRI center is informed and corrective actions are taken because it is highly likely more spikes will be produced by the system (QA protocol). As the manuscript focuses more on QC decisions than QA aspects, we've updated the text accordingly:

Edit: **Original (p. 2, ll. 50–53):**

> Understanding how defacing alters the initial quality ~~assurance (QA)~~ checkpoint of the research workflow [7] is necessary because there is compelling evidence that data showing specific artifacts or insufficient overall quality introduce bias into the results of analyses, raising questions about their validity [8–10].

Revised (p. 2, ll. 53–56):

> Understanding how defacing alters the initial quality checkpoint of the research workflow [7] is necessary because there is compelling evidence that data showing specific artifacts or insufficient overall quality introduce bias into the results of analyses, raising questions about their validity [8–10].

Edit: **Original (p. 2, ll. 56–58):**

> However, the reliability of automated ~~QC~~ alternatives is limited mainly due to site-effects [11], which leads to implementing ~~QA/~~QC manually by screening the imaging data on a one-by-one basis.

Revised (p. 3, ll. 59–64):

> [Sentence completely changed in response to R#1.16]

Edit: **Original (p. 2, ll. 61–63):**

> Large consortia have also made substantial investments in this critical task and have generated valuable contributions to QA~~/QC~~ protocols, e.g., the Human Connectome Project [14], or the INDI initiative (QAP [15]).

Revised (p. 3, ll. 67–69):

> Large consortia have also made substantial investments in this critical task and have generated valuable contributions to quality assurance (QA) protocols and corresponding QC criteria, e.g., the Human Connectome Project [14], or the INDI initiative (QAP [15]).

Edit: **Original (p. 3, ll. 68–71):**

> Moreover, because ~~the QA/~~QC is onerous, many teams have attempted automation, either by defining no-reference (that is, no ground truth is available) IQMs that can be used to learn a machine predictor [6,15,17], or by training deep models directly on 3D images [18].

Revised (p. 3, ll. 75–77):

Moreover, because manual QC is onerous, many teams have attempted automation, either by defining no-reference (that is, no ground truth is available) IQMs that can be used to learn a machine predictor [6,15,17], or by training deep models directly on 3D images [18].

Edit: **Original (p. 3, l. 76):**

The present registered report builds on our exploratory analysis to confirm that four trained human raters made systematically varying ~~QA~~ decisions when rating the same images depending on whether the have been defaced.

Revised (p. 3, l. 83):

The present registered report builds on our exploratory analysis to confirm that four trained human raters made systematically varying QC decisions when rating the same images depending on whether the have been defaced.

Edit: **Original (p. 3, l. 77):**

Regarding automated ~~QA~~, this paper shows that differences in IQMs extracted with MRIQC before and after defacing are not meaningful.

Revised (p. 3, l. 84):

Regarding automated QC, this paper shows that differences in IQMs extracted with MRIQC before and after defacing are not meaningful.

Edit: **Original (p. 3, l. 82):**

This pre-registered report was set out to confirm whether defacing alters the manual and automatic ~~QA~~ of T1w images for the healthy human brain—implemented with *MRIQC*.

Revised (p. 4, l. 92):

This pre-registered report was set out to confirm whether defacing alters the manual and automatic QC of T1w images for the healthy human brain—implemented with *MRIQC*.

Edit: **Original (p. 5, l. 151):**

To assess the intra-rater effects on ~~QA~~,

Revised (p. 6, l. 169):

To assess the intra-rater effects on QC,

Edit: **Original (p. 7, l. 237):**

Defacing impact on automatic ~~QA~~ was evaluated based on

Revised (p. 9, l. 273):

Defacing impact on automatic QC was evaluated based on

Edit: **Original (p. 11, l. 344):**

Second, we considered that ~~QA/~~QC is more concerned with sensitivity

Revised (p. 12, l. 386):

Second, we considered that QC is more concerned with sensitivity

Edit: **Original (p. 16, l. 495):**

This bias is present in all datasets (as QA attempts to minimize data exclusion on the grounds of insufficient quality),

Revised (p. 19, l. 573):

This bias is present in all datasets (as QA/QC attempts to minimize data exclusion on the grounds of insufficient quality),

Edit: **Original (p. 16, ll. 498–500):**

For this reason, we argue that improving the automated ~~QA/~~QC of T1w images will require that all available data be shared, including samples discarded because they met the study's exclusion criteria.

Revised (p. 19, ll. 576–578):

For this reason, we argue that improving the automated QC of T1w images will require that all available data be shared, including samples discarded because they met the study's exclusion criteria.

Edit: **Original (p. 16, ll. 500–501):**

Doing so will enable the development of more sensitive and specific ~~QA/~~QC tools that are robust to site effects.

Revised (p. 19, ll. 578–579):

Doing so will enable the development of more sensitive and specific QC tools that are robust to site effects.

Edit: **Original (p. 16, ll. 503–504):**

Factors like the type of analysis or the region of interest in addressing the research question influence these QA/QC ~~decisions~~ [23].

Revised (p. 19, ll. 581–582):

Factors like the type of analysis or the region of interest in addressing the research question influence these QA/QC protocols [23].

Edit: **Original (p. 16, ll. 511–513):**

Our pre-registration failed to set corresponding ~~QA/~~QC criteria to assess the raters' performance and anticipate countermeasures.

Revised (p. 19, ll. 589–591):

Our pre-registration failed to set corresponding QC criteria to assess the raters' performance and anticipate countermeasures.

Edit: **Original (p. 17, ll. 559–560):**

~~We argue that sharing QA/QC materials from nondefaced images can help mitigate these issues while ensuring participant privacy.~~

Revised (p. 21, ll. 649–658):

[Sentence edited previously—see response to R#1.4]

R#1.7:

Assuming "eye-spillover" is ghosting from eye motion?

AR: We appreciate that the reviewer highlighted that our use of the term "eye-spillover" is insufficiently described. Here, we continue the discussion of this point that was deferred from the response to comment R#1.5. As indicated on R#1.5, we avoided the vague and idiosyncratic terminology of "eye spillover" and have provided further explanations addressing the reviewer's comment within the caption of Figure 1. This edit of the caption also includes the details about the background visualization requested in comment R#1.9.

Edit: **Original (p. 2, Figure 1):**

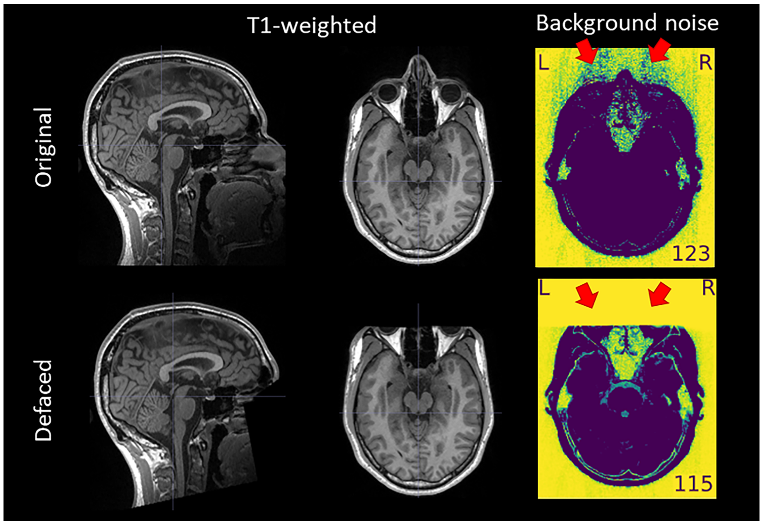

Figure 1. An example of T1w image before and after defacing. Defacing is typically

implemented by zeroing the voxels around the face. The background noise visualization ~~is~~ extracted from the MRIQC visual report ~~and illustrates that eye spillover is one example of information key in evaluating image quality that is removed by defacing.~~

Revised (p. 2, Figure 1):

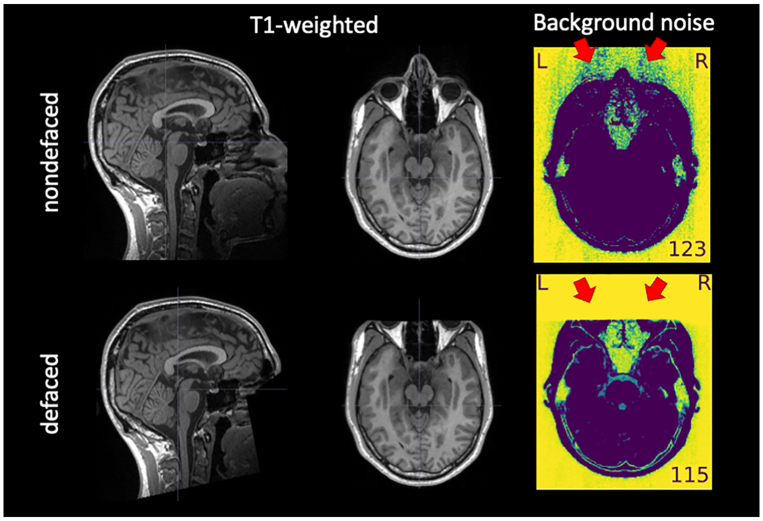

Figure 1. An example of T1w image before and after defacing. Defacing is typically implemented by zeroing the voxels around the face (left-hand side, panel “T1-weighted”). The “Background noise” panel shows two visualizations extracted from the *MRIQC*’s visual report, in which a window is applied to select the lowest intensities and then they are inverted to enhance patterns in the background. The red arrows indicate aliases along the anterior-posterior axis—which had the lowest bandwidth in the example—produced by eye motion. These aliases are straightforward to notice in front of the ocular globes in the “Background noise” panel of the “nondefaced” image because of the absence of signal sources. This aliasing also spreads in the opposite direction, overlapping brain tissue. However, this overlap is often very hard to notice against the signal of interest within the brain. The corresponding “defaced” version of the “Background noise” panel shows how defacing eliminates valuable information for quality assessment.

While the reviewer understandably interprets this artifact as a consequence of excessive eye movement, “eye spillover” can be a proxy for different issues. It would be unsurprising to find “eye spillover” in the images of a study with only healthy, compliant, and collaborative volunteers if the imaging parameters were “too ambitious”. In this case, eye spillover becomes “the canary in the coal mine”: while subjects moved their eyes “just as-much/as-little as the average, collaborative subject,” all images across subjects in the study are affected by subtle eye spillover. If researchers chose an excessively short readout time, then the bandwidth of the phase encoding direction is too narrow and every relatively fast motion (e.g., bloodstream pulsations) will undermine image quality. As now included in the manuscript, the eye aliases will be widespread over the image through the axis with the smallest bandwidth after reconstruction.

R#1.8:

I don't follow this statement - and it makes it sound like the main result has been caused by poor study design? I think it needs a better explanation L331 "Our variation of the BA plot revealed that images rated highly in the nondefaced condition were, by design, very unlikely to receive a higher rating in the defaced condition, which we did not anticipate at pre-registration time."

AR: We acknowledge that the phrasing (ll. 331–332) is confusing. More importantly, this particular statement seems out of place as it makes an interpretation of an additional visualization we made in addition to the pre-registered plan of using "traditional" BA plots. We have removed this sentence, as the already referenced Fig. S2, the caption of Fig. 2, and the discussion make a much better description of this matter.

Edit: **Original (p. 10, ll. 329–332):**

> A side-by-side comparison between the standard BA plots and our "optimized" BA plot is shown in Fig. S2. ~~Our variation of the BA plot revealed that images rated highly in the nondefaced condition were, by design, very unlikely to receive a higher rating in the defaced condition, which we did not anticipate at pre-registration time.~~

Revised (p. 12, ll. 371–375):

> A side-by-side comparison between the standard BA plots and our "optimized" BA plot is shown in Fig. S2.

R#1.9:

Explain how your "Background noise" image in Figure 1 has been made (appears to be by thresholding but should be included in the caption)

AR: We acknowledge this visualization was not clearly explained and thank the reviewer for this comment. Additionally, we noticed the label "Original" is inconsistent with nomenclature throughout the paper. We have fixed the label to conform with the term used elsewhere ("nondefaced"). We have edited Figure 1 and its corresponding caption correspondingly to this and the previous comment (see R#1.7).

R#1.10:

Include a comment at the start of the data section L92 that the lettered footnotes are related to protocol deviations which can be found at the end.

AR: Absolutely:

Edit: **Original (p. 3, ll. 79–80):**

> **Methods**
> Hypotheses

Revised (p. 3, ll. 87–90):

> **Methods**

The lettered footnotes in this section indicate deviations from the Stage 1 protocol and are reported in the "Protocol Deviations" subsection.

Hypotheses

R#1.11:

The bias - defined L421 - should be defined on first use not after, i.e. before L415 - and a quick note on how it's computed (assuming it is the average of non-deface- defaced score for all images that rater scored?)

AR: We thank the reviewer for noticing this issue, which led to the necessary changes introduced in the initial General comment. The reviewer is right in their assumption. We have updated the manuscript accordingly, improving the nomenclature and including the definition of the bias $\mathbb{E}[\Delta_{\text{ndef-def}}]$, as well as the calculation of LoA and 95% CI (see the General comment) as—befittingly—requested by the reviewer.

The revised manuscript also includes some downstream edits derived from the previous changes:

Edit: **Original (p. 13, ll. 414–415):**

Our most experienced rater (Rater 1) presented the highest bias~~, defined as nondefaced minus defaced $\Delta_{\text{ndef-def}}$~~ = -0.12 (Table S2), (...)

Revised (p. 14, ll. 457–458):

Our most experienced rater (Rater 1) presented the highest bias $\mathbb{E}[\Delta_{\text{ndef-def}}]$=–0.12 (Table S2), (...)

Edit: **Original (p. 13, l. 416):**

a more junior rater (Rater 4) barely modified their quality assessment (~~$\Delta_{\text{ndef-def}}$ = -0.01~~).

Revised (p. 14, ll. 459–460—also fixing a rounding error):

a more junior rater (Rater 4) barely modified their quality assessment ($\mathbb{E}[\Delta_{\text{ndef-def}}]$=-0.02).

Edit: **Original (p. 13, ll. 421–424):**

~~The bias (nondefaced – defaced, $\Delta_{\text{ndef-def}}$)~~ was consistently negative across all raters, implying that defaced images were, on average, scored with a higher rating. ~~Yet,~~ this bias was ~~not~~ significantly ~~consistent because the 95% limits of agreement on the BA plot pooling all raters contained zero,~~ as shown in Fig. ~~S~~2.

Revised (pp. 14–15, ll. 464–468):

$\mathbb{E}[\Delta_{\text{ndef-def}}]$ was consistently negative across all raters, implying that defaced images were, on average, scored with a higher rating. This bias was significant for Raters 1 and 3, and all raters aggregated as shown in Fig. 2 and comprehensively reported in Table S2.

Edit: **Original (p. 14, l. 444):**

Rater 4 also displayed a lower spread in quality assessments (see Fig. S5).

Revised (p. 16, ll. 503–504):

Rater 4 also displayed a lower spread in quality assessments, which translated into the narrowest 95% LoA (Fig. 2).

Edit: **Original (p. 15, l. 479):**

the largest bias $\Delta_{\text{ndef-def}}$ = -0.12 (Table S2)

Revised (p. 17, l. 550):

the largest bias $\mathbb{E}[\Delta_{\text{ndef-def}}]$=-0.12 (Table S2)

Edit: **Original (p. 13, Figure 2):**

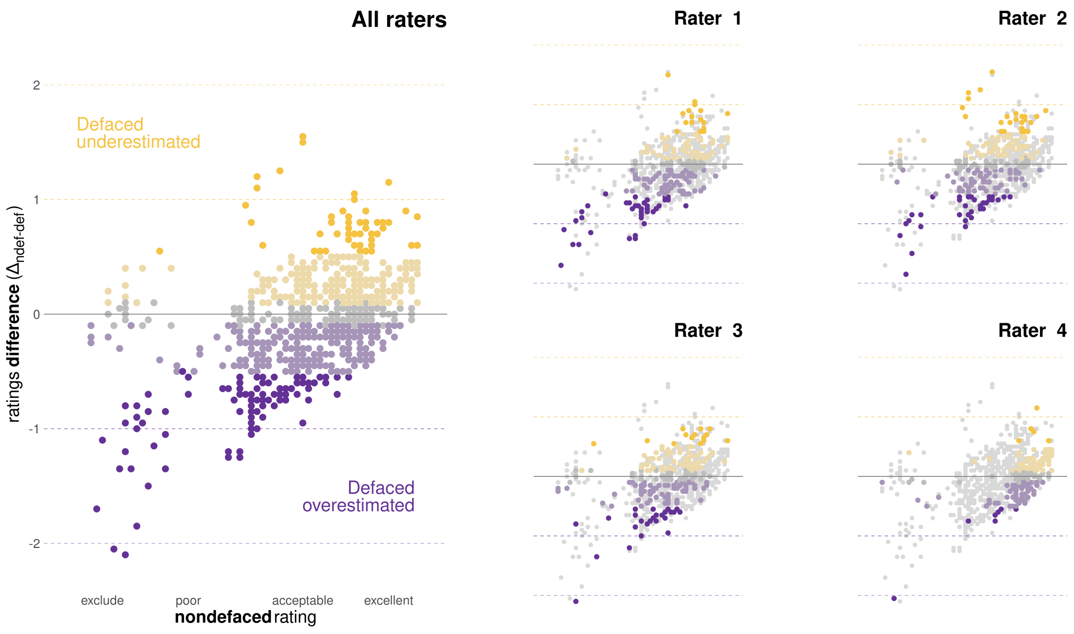

Figure 2. Defacing biases human assessment of image quality, particularly when image quality is low. Nondefaced images with low ratings were evaluated as similar or better quality after defacing, whereas nondefaced images with high ratings were assessed as similar or lower quality. This variability at the extremes is explained by our rating scale being constrained to the range [1,4]. More interestingly, we observe that lower ratings in the nondefaced condition vary the most. This suggests that when more pronounced artifacts were present, defacing removed critical information needed to evaluate image quality. The y-axis corresponds to the defaced rating subtracted from the nondefaced rating. Reliable ratings (i.e., ratings with absolute differences lower than 0.1) are colored in grey and ratings that were overestimated or underestimated after defacing are colored in purple or yellow,

~~respectively. The points are plotted with lower luminosity if their absolute difference is below 0.5, indicating a moderate defacing bias.~~

Revised (p. 15, Figure 2):

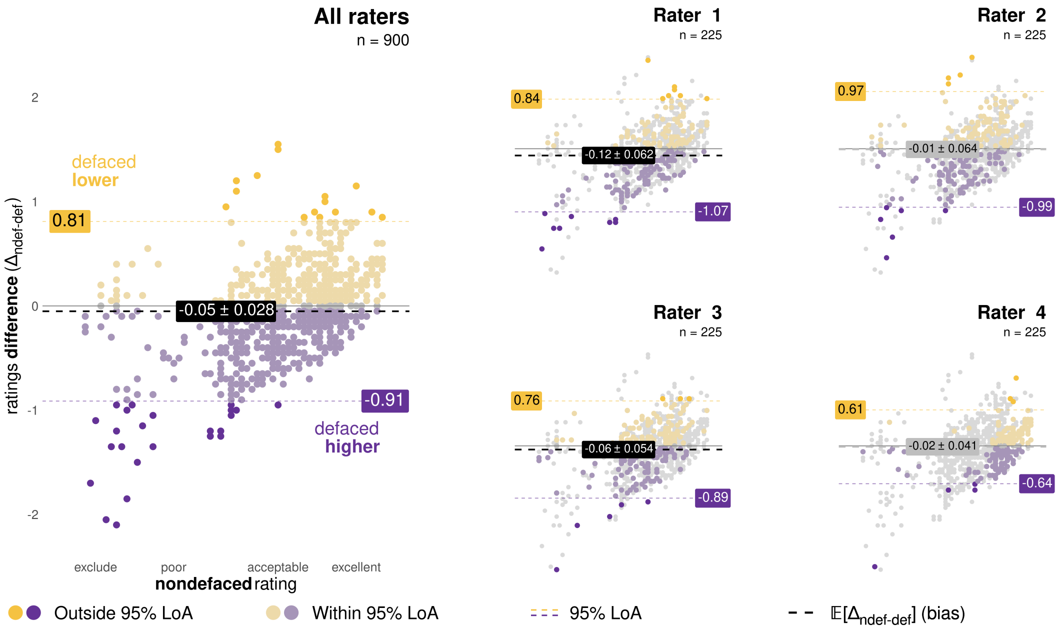

Figure 2. Defacing biases human assessment of image quality, particularly when image quality is low. We examined biases with an "optimized" version of the BA plot, in which the x-axis represents the rating assigned to the nondefaced version of an image. Corresponding "standard" BA plots—in which the x-axis shows the average of the two ratings [37]—are reported in Fig. S2. Rating pairs where the defaced image's quality was underestimated with respect to the nondefaced ($\Delta_{\text{ndef-def}} > 0$) are represented in yellow. Conversely, pairs where the defaced image's quality was overestimated ($\Delta_{\text{ndef-def}} < 0$) are in purple. Pairs within the 95% LoA are represented with dim colors, and the LoA boundaries are indicated with dashed colored lines annotated with their value. For example, the LoA for all raters pooled together was [-0.91, 0.81] (left panel). Finally, the bias $\mathbb{E}[\Delta_{\text{ndef-def}}]$ is represented by a gray or black dashed line with a label reporting value and their corresponding 95% CI interval (parametric estimation). All raters displayed 95% LoA exceeding one unit, indicating that defacing introduces large variability in human assessments. All raters had negative—albeit small—biases, indicating that they systematically rated defaced images higher. However, these biases were statistically significant only for Raters 1 and 2, as well as the four raters aggregated together—indicated by the bias label and line colored in black. Relevant statistics (bias, LoA, 95% CI) are reported in Table S2, and the full report of statistics, including 95% CI intervals calculated both by parametric and non-parametric means are distributed as tab-separated-values files on the corresponding GitHub repository.

R#1.12:

Figure 2 - "Defaced Underestimated" label would be clearer as "Defaced rated worse" (or "lower"), and similarity - "Defaced overestimated" might be clearer as "Defaced rated better" (or "higher").

AR: We have updated the figure accordingly (see changes on the figure in the response to comment R#1.11)

R#1.13:

Abstract - L17 - "requirement" should be "step" since requirement might be different for dependent on the study.

AR: We thank the reviewer for this suggestion.

Edit: **Original (p. 1, l. 17):**

A critical ~~requirement~~ before data-sharing of human neuroimaging is removing facial features to protect individuals' privacy.

Revised (p. 1, l. 17):

A critical step before data-sharing of human neuroimaging is removing facial features to protect individuals' privacy.

R#1.14:

L39 - what type of automated analysis method fail? and what does "failed in execution" mean? Grammatical error in there too.

AR: We thank the reviewer for suggesting those clarifications and have edited the manuscript accordingly. These edits also address the following comment R#1.15.

Edit: **Original (pp. 1–2, ll. 38–41):**

De Sitter et al. [4] ~~showed that downstream~~ automated ~~analysis methods failed in execution up to 19% of the cases after defacing, as opposed to 2% on nondefaced counterparts~~. They also reported systematic differences between the same processing with and without defacing in several outcomes of interest ~~in neurodegeneration studies~~.

Revised (pp. 1–2, ll. 38–45):

De Sitter et al. [4] investigated the undesired effects of defacing on automated workflows in neurodegeneration studies. Failure rates—i.e., the percentage of inputs for which workflows did not finish successfully—were substantially larger on defaced inputs (19%) compared to their nondefaced counterparts (2%). They also reported systematic differences between the same processing with and without defacing in several outcomes of interest, such as gray- and white-matter volume and cortical thickness.

R#1.15:

L41 - outcomes of interest such as? GM volume? Give example

AR: We have updated accordingly, as reported in the previous comment.

R#1.16:

L57 - replace "is" with "may be"

AR: We thank the reviewer for pointing out a confusing phrasing. We have edited the sentence to avoid the multiplicity of interpretations it currently seems to elicit.

Edit: **Original (p. 2, ll. 56–58):**

> ~~However, the reliability of automated QC alternatives is limited mainly due to site-effects [11], which leads to implementing QA/QC manually by~~ screening ~~the~~ imaging data on a one-by-one basis.

Revised (p. 3, ll. 59–64):

> Our previous efforts to automate QC by learning *MRIQC*'s IQMs [6, 11] demonstrated that site-effects imposed the largest limitation on the performance. The reportedly modest reliability of automated QC alternatives currently available explains that QA/QC continues to require the screening of imaging data on a one-by-one basis.

R#1.17:

L93 "confirmatory" here maybe lessens the importance of the work carried out?

AR: We thank the reviewer for this suggestion.

Edit: **Original (p. 3, l. 93):**

> This ~~confirmatory~~ analysis was based on the publicly available IXI dataset [20], (...)

Revised (p. 4, l. 103):

> This analysis was based on the publicly available IXI dataset [20], (...)

R#1.18:

L108 PyDeface because it <currently> presents the highest success rate...

AR: We thank the reviewer for this suggestion.

Edit: **Original (p. 4, l. 108):**

> We chose *PyDeface* because it presents the highest success rate at removing facial features (...)

Revised (p. 4, l. 118):

We chose *PyDeface* because it currently presents the highest success rate at removing facial features (...)

R#1.19:

L111 the sentence starting "Thus. . . " could be reworded to avoid double negatives and be easier to understand. Subsequently the sentence on L113 starting "Under our hypotheses . . . could be re-worded as I don't quite follow what is trying to be said

AR: We agree with the reviewer that these sentences' clarity needed improvement and thank the reviewer for this suggestion.

Edit: **Original (p. 4, ll. 111–113):**

Fatal failure of *PyDeface* was the only exclusion criterion. ~~Thus, no images were excluded if not fully defaced—meaning that *PyDeface* was ineffective, and the image retained facial features such as sections of the eyes and the surrounding background[d]. Under our hypotheses, such images were expected to be more consistent between conditions for humans and machines and potentially contribute to reducing effect sizes. No image led to the fatal failure of *PyDeface* nor retained partial facial features[d].~~

Revised (pp. 4–5, ll. 120–130):

Fatal failure of *PyDeface* was the only exclusion criterion. Because all images were successfully processed, no images were dropped from the analysis for this reason. To avoid exclusion bias that could artificially inflate the effect size, images showcasing low-quality defacing, such as those partially retaining facial features—e.g., the eyes,— were included in the analysis[d]. These cases were expected to be more similar between defaced and nondefaced conditions, potentially attenuating differences in human ratings and IQMs.

R#1.20:

IQM seems to be first defined in the conclusion

AR: We have confirmed that the particular acronym (IQM) was defined on p. 2, l. 44, at its first occurrence. We have removed the re-definition of the acronym in the conclusion:

Edit: **Original (p. 17, l. 556):**

MRIQC's automatically-extracted ~~image quality metrics (~~IQMs~~)~~,

Revised (p. 21, l. 653):

MRIQC's automatically-extracted IQMs,

Minor corrections not prompted by the reviewer

Edit: **Original (p. 7, l. 215):**

η = 0.045 (i.e., a medium effect size) following Equation S2.

Revised (p. 7, l. 231):

η = 0.045 (i.e., a medium effect size) following Equation S1.

Edit: **Original (p. 7, l. 221):**

With G*Power (Fig. S7), we also estimated the noncentrality parameter λ

Revised (p. 7, l. 237):

With G*Power (Fig. S11), we also estimated the noncentrality parameter λ

Edit: **Original (p. 8, l. 256):**

f=0.16 (see Equations S5 and S6 for its computation).

Revised (p. 9, l. 292):

f=0.16 (see Equations S4 and S5 for its computation).

Edit: **Original (p. 8, l. 262):**

(see Equation S4 for the conversion of Cohen's d to Cohen's f).

Revised (p. 9, l. 298):

(see Equation S3 for the conversion of Cohen's d to Cohen's f).

Edit: **Original (p. 14, l. 447):**

The same observation is visible on the BA plots (Figs. 4 and S3).

Revised (p. 16, l. 507):

The same observation is visible on the BA plots (Figs. 2 and S2).

Edit: **Original (p. 14, l. 457):**

(η = 0.0047, f = 0.069; see Equation S6).

Revised (p. 16, l. 517):

(η = 0.0047, f = 0.069; see Equation S1).

We noticed a factual error in the caption of Table 4. Specifically, the effect size related to defacing (η_p^2=0.0012) is larger than the referenced effect size related to the site (η_p^2=7.9 $\cdot$ 10^{-4}). Given this, and considering that the sentence in question did not add new information to the caption, we decided to remove it to avoid any potential confusion. The caption also contained an incorrect cross-reference to Equation S5, which has been fixed.

Edit: **Original (p. 15, Table 4, ll. 464–472):**

Table 4. Results of repeated-measures MANOVA on the projected IQMs. We did not find an effect of the site nor an effect of defacing on the principal components extracted from the IQMs. However, applying the IQMs standardization and PCA separately per site mitigated the site effects, revealing a defacing bias. The much smaller effect size associated with the defaced variable compared to the site variable in the rm-MANOVA, which included a single standardization and PCA across all sites, suggests that the larger confounding effect of the site likely overshadowed the impact of defacing. Despite being significant, the defacing bias is associated with a negligible effect size. Effect size is reported as partial eta-squared (η_p^2) and was computed with the function *F_to_eta2* from the *R* package *effectsize* [46], which implements the formula given in Equation S5 with df = numDF and df_error = denDF. p_{FDR} corresponds to p-values controlled for false discovery rate (FDR).

Revised (p. 17, Table 4, ll. 530–538):

Table 4. Results of repeated-measures MANOVA on the projected IQMs. We did not find an effect of the site nor an effect of defacing on the principal components extracted from the IQMs. However, applying the IQMs standardization and PCA separately per site mitigated the site effects, revealing a defacing bias. Despite being significant, the defacing bias is associated with a negligible effect size. Effect size is reported as partial eta-squared (η_p^2) and was computed with the function *F_to_eta2* from the *R* package *effectsize* [46], which implements the formula given in Equation S4 with df = numDF and df_error = denDF. p_{FDR} corresponds to p-values controlled for false discovery rate (FDR).

Edit: **Original (p. 2, ll. 75-78):**

The present registered report builds on our exploratory analysis to confirm that four trained human raters make systematically varying QC decisions when rating the same images depending on whether they have been defaced. Regarding automated QC, this paper shows that differences in IQMs extracted with *MRIQC* before and after defacing are not meaningful.

Revised (p. 3, ll. 82–86):

The present registered report built on our exploratory analysis and confirmed that four trained human raters made systematically varying QC decisions when rating the same images depending on whether they had been defaced. Regarding automated QC, this paper showed that differences in IQMs extracted with *MRIQC* before and after defacing were not meaningful.

Edit: **Original (p. 8, l. 244):**

was applied to IQMs to the entire dataset PCA

Revised (p. 9, l. 280):

was applied to IQMs to the entire dataset. PCA

Edit: **Original (p. 12, l. 411):**

the difference amongst the highest ratings remained within 0.5 points.

Revised (p. 14, l. 453):

the difference amongst the highest ratings remained within 0.5 units.
