# Supplementary material for: Removing facial features from structural MRI images biases visual quality assessment
Source: PLoS Biol. 2025 Apr 30;23(4):e3003149. doi: 10.1371/journal.pbio.3003149 (PMC12143891; doi:10.1371/journal.pbio.3003149)
Supplement: Attachment — Submitted filename: PBIOLOGY-D-24-02964R3-response_letter.pdf [file pbio.3003149.s015.pdf]

Authors' Response to Reviews of

C. Provins et al. – "Removing facial features from structural MRI images biases visual quality assessment"

AR: Authors' Response

We thank the Academic Editor and the PLOS Biology team for their guidance and final suggestions. We are pleased to submit what we anticipate to be our final revision of the manuscript, having addressed all outstanding policy and data requests in full.

In this response, we reproduce all changes made to our submission, marked up with the *Edit* keyword. Page and line numbers of the "Original" text cross-reference the submitted version **without** markup of tracked changes. Page and line numbers of the "Revised" text cross-reference the new document **with** markup of tracked changes. For example:

Edit: **Original (p. N, ll. aaa–bbb):**

> Text in the ~~submitted~~ manuscript—version with~~out~~ track-changes markup,—with ~~deletions marked up by a strike-through line~~.

Revised (p. M, ll. yyy–zzz):

> Text in the revised manuscript—version with track-changes markup,—with additions marked up in blue-color text.

Comment #1:

We would like to suggest a different title to improve its accessibility for our broad audience: ""*Removing facial features from structural MRI images biases visual quality assessment*"

AR: We agree this is a better title.

Edit: **Original (p. 1, l. 1—Title):**

> ~~Defacing biases visual quality assessments of structural MRI~~

Revised (p. 1, l. 1—Title):

> Removing facial features from structural MRI images biases visual quality assessment

Comment #2:

Please include the approval/license number of the ethical approval for experiments (if required) and include information in the Methods section whether the study has been conducted according to the principles expressed in the Declaration of Helsinki. Please also specify whether the participants provided written or oral consent.

AR: We have updated the methods section as follows:

Edit: **Original (p. 4, ll. 109–111):**

> The local ethics committee ~~has~~ approved the processing of nondefaced images. This study does not attempt to re-identify the participants nor facilitate in any way such efforts[c].

Revised (p. 4, ll. 112–117):

> The local ethics committee (Swiss BASEC—Business Administration System for Ethics Committees) approved the processing of nondefaced images (project 2022-00360). This study involved only the reuse of publicly shared data. The original data were collected in accordance with the Declaration of Helsinki, with written informed consent obtained by the original investigators. This study did not attempt to re-identify the participants nor facilitate in any way such efforts[c].

Comment #3: DATA POLICY

You may be aware of the PLOS Data Policy, which requires that all data be made available without restriction: http://journals.plos.org/plosbiology/s/data-availability. For more information, please also see this editorial: http://dx.doi.org/10.1371/journal.pbio.1001797
Note that we do not require all raw data. Rather, we ask that all individual quantitative observations that underlie the data summarized in the figures and results of your paper be made available in one of the following forms:
1) Supplementary files (e.g., excel). Please ensure that all data files are uploaded as 'Supporting Information' and are invariably referred to (in the manuscript, figure legends, and the Description field when uploading your files) using the following format verbatim: S1 Data, S2 Data, etc. Multiple panels of a single or even several figures can be included as multiple sheets in one excel file that is saved using exactly the following convention: `S1_Data.xlsx` *(using an underscore).*
2) Deposition in a publicly available repository. Please also provide the accession code or a reviewer link so that we may view your data before publication.
Regardless of the method selected, please ensure that you provide the individual numerical values that underlie the summary data displayed in the following figure panels as they are essential for readers to assess your analysis and to reproduce it: 3, S3, S5, S6, S12, S13 and S20.
NOTE: the numerical data provided should include all replicates AND the way in which the plotted mean and errors were derived (it should not present only the mean/average values).
Please also ensure that figure legends in your manuscript include information on where the underlying data can be found, and ensure your supplemental data file/s has a legend.
Please ensure that your Data Statement in the submission system accurately describes where your data can be found.

AR: We fully acknowledge and comply with the PLOS Data Policy and have ensured that all data are made available without restriction, in both tabular and visual formats as specified. For tabular data, we have implemented option (1), and only for the visual reports—which entails a voluminous tree of HTML documents and SVG images—we have opted for option (2) through FigShare.

All data underlying all figures in the main text and the Supplementary Materials has been included, with the sole deviation that we employed tab-separated-values (TSV) instead of the proprietary format of Microsoft Excel.

Figure legends have been updated as follows:

Edit: **Original (Fig. 2, p. 14, ll. 465–467):**

> Relevant statistics (bias, LoA, 95% CI) are reported in Table S2, and the full report of statistics, including 95% CI intervals calculated both by parametric and non-parametric means are distributed ~~as tab-separated-values files on the corresponding GitHub repository~~.

Revised (Fig. 2, pp. 14–15, ll. 471–475):

> Relevant statistics (bias, LoA, 95% CI) are reported in Table S2, and the full report of statistics, including 95% CI intervals calculated both by parametric and non-parametric means are distributed within the `S6_Data.tsv` supplementary data file. Source tabular data for the BA analysis and results in Table S2 are found within the `S1_Data.tsv` supplementary data file.

Edit: **Original (Fig. 3, p. 15, ll. 479–480):**

> Higher ratings displayed gaps within 0.5 units. ~~The same observation is visible on the BA plots~~ (Figs. 2 and S2).

Revised (Fig. 3, p. 15, ll. 487–489):

> Higher ratings displayed gaps within 0.5 units. BA plots support the same observation (Figs. 2 and S2). Source tabular data to generate this figure are found within the `S1_Data.tsv` supplementary data file.

We have correspondingly updated all the figure captions in the Supplementary Materials pointing to the specific data files.

We have also updated the Data and code availability accordingly, ensuring tabular data and visual reports are explicitly indicated. We have also removed a mention of a Web Service because it is cited with a reference in the main text.

Edit: **Original (p. 19, ll. 620–633):**

> **Data and code availability statement**
>
> All the new materials relating to this work were shared under suitable open licenses (Apache 2.0 for code and CC-BY for data, unless otherwise specified) before the Stage 2 report submission. The IXI dataset is available at https://brain-development.org/ixi-dataset/ under the Creative Commons CC BY-SA 3.0 license. All the code related to this study ~~and tabular data collected (manual ratings and the related covariates, as well as the IQMs extracted from defaced and nondefaced) are~~ openly shared under the Apache 2.0 license at https://github.com/TheAxonLab/defacing-and-qc-analysis. ~~The Web Service we implemented to collect the manual ratings in this study is available under the Apache 2.0 license at https://github.com/nipreps/qkay.~~ The presentation used in the training session can be accessed at https://osf.io/d57mb/, and the training reports are openly shared at https://github.com/TheAxonLab/defaci[illegible] and-qc-trainingsession. The MRIQC reports generated from the IXI dataset and used by the human raters in their assessment ~~are accessible via https://github.com/TheAxonLab/defacing-and-qc-ixi-reports~~ under the terms of the CC-BY-SA-4.0 license, as they are derived from the original IXI dataset.

Revised (pp. 19–20, ll. 629–655):

> **Data and code availability statement**
>
> All the new materials relating to this work were shared under suitable open licenses (Apache 2.0 for code and CC-BY for data, unless otherwise specified) before the Stage 2 report submission. The IXI dataset is available at https://brain-development.org/ixi-dataset/ under the Creative Commons CC-BY-SA 3.0 license. All the tabular source data—i.e., manual ratings and extracted IQMs,—as well as the outputs of analyses are made available with this manuscript as supplementary data files and under the same license as the manuscript itself. Specifically, files `S1_Data.tsv` and `S2_Data.tsv` contain the source data, files `S3_Data.tsv`, `S4_Data.tsv`, and `S5_Data.tsv` the PCA projections for each of the three corresponding analyses and finally, files `S6_Data.tsv` and onwards contain the analyses' outputs. The *MRIQC* reports generated from the IXI dataset and used by the human raters in their assessment have been deposited on *FigShare* (doi:10.6084/m9.figshare.28684367) under the terms of the CC-BY-SA-4.0 license, as they are derived from the original IXI dataset. The dataset is accessible under version control with *DataLad* at https://github.com/TheAxonLab/defacing-and-qc-ixi-reports. All the code related to this study is openly shared under the Apache 2.0 license at https://github.com/TheAxonLab/defacing-and-qc-analysis. The presentation used in the training session can be accessed at https://osf.io/d57mb/, and the training reports are openly shared at https://github.com/TheAxonLab/defacing-and-qc-trainingsession.

> **Comment #4: CODE POLICY**
>
> *Per journal policy, if you have generated any custom code during the course of this investigation, please make it available without restrictions. Please ensure that the code is sufficiently well documented and reusable, and that your Data Statement in the Editorial Manager submission system accurately describes where your code can be found.*
> *Please note that we cannot accept sole deposition of code in GitHub, as this could be changed after publication. However, you can archive this version of your publicly available GitHub code to Zenodo. Once you do this, it will generate a DOI number, which you will need to provide in the Data Accessibility Statement (you are welcome to also provide the GitHub access information). See the process for doing this here: https://docs.github.com/en/repositories/archiving-a-github-repository/referencing-and-citing-content*

AR: We have deposited the submitted version of the code on Zenodo, tagged the specific commit, and updated our Data and code availability statement as follows (including the changes from the previous comment):

Edit: **Original (p. 19, ll. 624–626):**

> All the code related to this study ~~and tabular data collected (manual ratings and the related covariates, as well as the IQMs extracted from defaced and nondefaced) are~~ openly shared under the Apache 2.0 license at https://github.com/TheAxonLab/defacing-and-qc-analysis.

Revised (p. 20, ll. 643–648):

> All the code related to this study is openly shared under the Apache 2.0 license at https://github.com/TheAxonLab/defa[illegible] and-qc-analysis. The specific commit in the *Git* history of the repository containing the code

to produce all the results included in this manuscript was tagged and deposited in Zenodo (doi:10.5281/zenodo.15103450).

Comment #5:

Please move all references from the supplementary information to the main reference list.

AR: References have been moved as requested and the Supplementary Materials document updated accordingly.

Edit: **Original (p. 25, l. 790):**

[end of main text's references]

Revised (pp. 25–26, ll. 816–853):

Supplementary References

S1. Benjamini, Yoav, and Yosef Hochberg. 1995. "Controlling the False Discovery Rate: A Practical and Powerful Approach to Multiple Testing." Journal of the Royal Statistical Society. Series B (Methodological) 57 (1): 289–300.

S2. Cicchetti, D. V., and S. A. Sparrow. 1981. "Developing Criteria for Establishing Interrater Reliability of Specific Items: Applications to Assessment of Adaptive Behavior." American Journal of Mental Deficiency 86 (2): 127–37.

S3. Cohen, Jacob. 1988. Statistical Power Analysis for the Behavioral Sciences. 2nd ed. New York: Routledge. https://doi.org/10.4324/9780203771587.

S4. Faul, Franz, Edgar Erdfelder, Axel Buchner, and Albert-Georg Lang. 2009. "Statistical Power Analyses Using G*Power 3.1: Tests for Correlation and Regression Analyses." Behavior Research Methods 41 (4): 1149–60. https://doi.org/10.3758/BRM.41.4.1149. Gamer, Matthias, Jim Lemon, and Ian Fellows Puspendra Singh. 2019. "Irr: Various Coefficients of Interrater Reliability and Agreement." https://CRAN.R-project.org/package=irr.

S5. Gorgolewski, Krzysztof J., Joke Durnez, and Russell A. Poldrack. 2017. "Preprocessed Consortium for Neuropsychiatric Phenomics Dataset." F1000Research. https://doi.org/10.12688/f1000research.11964.2.

S6. Gudicha, Dereje W., Verena D. Schmittmann, and Jeroen K. Vermunt. 2016. "Power Computation for Likelihood Ratio Tests for the Transition Parameters in Latent Markov Models." Structural Equation Modeling: A Multidisciplinary Journal 23 (2): 234–45. https://doi.org/10.1080/10705511.2015.[illegible]

S7. Li, Qizhai, Junjian Zhang, and Shuai Dai. 2009. "On Estimating the Non-Centrality Parameter of a Chi-Squared Distribution." Statistics & Probability Letters 79 (January):98–104. https://doi.org/10.1016/j.spl.2008.07.025.

S8. Mortamet, Bénédicte, Matt A. Bernstein, Clifford R. Jack, Jeffrey L. Gunter, Chadwick Ward, Paula J. Britson, Reto Meuli, Jean-Philippe Thiran, Gunnar Krueger, and Alzheimer's Disease Neuroimaging Initiative. 2009. "Automatic Quality Assessment in Structural Brain Magnetic Resonance Imaging." Magnetic Resonance in Medicine 62 (2): 365–72. https://doi.org/10.1002/mrm.21992.

S9. Provins, Céline, Eilidh MacNicol, Saren H. Seeley, Patric Hagmann, and Oscar Esteban.

2023. "Quality Control in Functional MRI Studies with MRIQC and fMRIPrep." Frontiers in Neuroimaging 1. https://doi.org/10.3389/fnimg.2022.1073734.

S10. R Core Team. 2021. "R: The R Project for Statistical Computing." 2021. https://www.r-project.org/.

S11. Shapiro, S. S., and M. B. Wilk. 1965. "An Analysis of Variance Test for Normality (Complete Samples)†." Biometrika 52 (3–4): 591–611. https://doi.org/10.1093/biomet/52.3-4.591.

S12. Wilk, M. B., and R. Gnanadesikan. 1968. "Probability Plotting Methods for the Analysis of Data." Biometrika 55 (1): 1–17. https://doi.org/10.2307/2334448.

Comment #6:

Please include a link to the OSF repository where the Stage 1 IPA manuscript can be found.

AR: We have updated the first sentence of the Methods section as follows:

Edit: **Original (p. 3, ll. 83–84):**

The lettered footnotes in this section indicate deviations from the Stage 1 ~~protocol~~ and are reported in the "Protocol Deviations" subsection.

Revised (p. 3, ll. 85–87):

The lettered footnotes in this section indicate deviations from the Stage 1 IPA (in-principle accepted) manuscript—doi:10.17605/OSF.IO/QCKET—and are reported in the "Protocol Deviations" subsection.

Comment #7:

Please mention somewhere in the manuscript (e.g. Acknowledgements) that the Stage 1 submission was reviewed and recommended by Peer Community in Registered Reports, with a link to the public recommendation and review history: https://rr.peercommunityin.org/articles/rec?id=346

AR: We have updated the Acknowledgements section accordingly:

Edit: **Original (p. 20, ll. 642–645):**

Acknowledgments

This work has been supported by the Swiss National Science Foundation—SNSF—(#185872, OE), the NIMH (RF1MH121867; OE, RP), and the Chan-Zuckerberg Initiative (EOSS5-266; OE). PH and YAG receive support from SNSF (#185897, PH), EFG receives support from SNSF (#10000706, EFG).

Revised (p. 20, ll. 665–670):

Acknowledgments

The Stage 1 protocol corresponding to this Registered Report was reviewed and recommended

by Peer Community in Registered Reports. The recommendation and review history are accessible at https://rr.peercommunityin.org/articles/rec?id=346.

This work has been supported by the Swiss National Science Foundation—SNSF—(#185872, OE), the NIMH (RF1MH121867; OE, RP), and the Chan-Zuckerberg Initiative (EOSS5-266; OE). PH and YAG receive support from SNSF (#185897, PH), EFG receives support from SNSF (#10000706, EFG).

Comment #8:

As you address these items, please take this last chance to review your reference list to ensure that it is complete and correct. If you have cited papers that have been retracted, please include the rationale for doing so in the manuscript text, or remove these references and replace them with relevant current references. Any changes to the reference list should be mentioned in the cover letter that accompanies your revised manuscript.

AR: We have revised the reference list and confirm it is complete and correct.
